# Relationship Between Gender and In-Hospital Morbidity and Mortality After Coronary Artery Bypass Grafting Surgery in an Iranian Population

**DOI:** 10.5812/cardiovascmed.4543

**Published:** 2012-11-01

**Authors:** Forouzan Yazdanian, Rasoul Azarfarin, Nahid Aghdaii, Soudabeh Jalali Motlagh, Zahra Faritous, Mostafa Alavi, Saeid Hosseini

**Affiliations:** 1 Department of Cardiac Anesthesiology, Rajaie Cardiovascular Medical and Research Center, Tehran University of Medical Sciences, Tehran, IR Iran; 2 Cardiovascular Research Center, Tabriz University of Medical Sciences, Tabriz, IR Iran; 3 Heart Valve Disease Research Center, Rajaie Cardiovascular Medical and Research Center, Tehran University of Medical Sciences, Tehran, IR Iran

## Abstract

**Background:**

Many previous studies have investigated the influence of gender on coronary artery bypass grafting surgery (CABG) outcomes. Despite the great volume of reports on this issue, it is still not clear whether it is the gender of the patient or pre-existing comorbid conditions that is the best predictor for the different outcomes seen between men and women. Multiple studies have shown that women are at higher risk of postoperative complications than men, particularly in the perioperative period.

**Objectives:**

The goal of this study was to determine whether sex differences exist in preoperative variables between men and women, and to evaluate the effect of gender on short-term mortality and morbidity after CABG in an Iranian population.

**Patients and Methods:**

Data were collected prospectively from 690 consecutive patients (495 men and 195 women) who underwent isolated CABG. Preoperative, intraoperative, and postoperative variables, major complications and death were compared between the male and female patients until hospital discharge using multivariate analysis.

**Results:**

Women were older (P = 0.020), had more diabetes (P = 0.0001), more obesity (P = 0.010), a higher New York Heart Association functional class (P = 0.030), and there was less use of arterial grafts (P = 0.016). Men had more tobacco smokers (P = 0.0001) and lower preoperative ejection fractions (EF) (P = 0.030). After surgery, women had a higher incidence of respiratory complications (P = 0.003), higher creatine kinase (CK) – MB levels (P = 0.0001), and higher inotropic support requirements (P = 0.030). They also had a higher incidence of decreased postoperative EF versus preoperative values (P = 0.020). The length of ICU stay, incidence of return to ICU and postoperative death, were similar between men and women. Nevertheless, after adjusting for age and diabetes, female gender was still independently associated with higher morbidity in patients over 50 years of age.

**Conclusions:**

Women had more risk factors, comorbidities, and postoperative complications. Women older than 50 years of age were at a higher risk of postoperative complications than men. This difference decreased with younger age. In-hospital mortality rates were not influenced by sex, as there was no difference found between the two groups (2.5% women vs. 2.2% men; P > 0.05).

## 1. Background

There has been an increase in coronary artery bypass grafting surgery (CABG) rates as it is an effective method for the management of patients with severe coronary artery disease (1), running parallel to this, many studies have investigated the influence of gender on CABG outcomes. Despite the great volume of reports on this issue, it is still unclear whether it is the gender or comorbid conditions in these patients that are the best predictors for the different outcomes between men and women. A number of studies have previously shown that women are at higher risk of postoperative complications than men, particularly in the perioperative period (1, 2). Different explanations exist for this observation. In some reports, variations between men and women are the responsible factor, women, compared with men; are older (3), have higher rates of comorbid conditions (2, 4), smaller coronary arteries (5, 6), more acute and unstable presentation or advanced stage of disease, referral bias (3, 7), and underutilization of the internal mammary artery (IMA) (8, 9). Numerous investigations have shown gender to be an independent risk factor for an adverse outcome (10-12). On the other hand, some investigators have found no significant difference in CABG operative mortality and morbidity rates between men and women (13, 14).

## 2. Objectives

The purpose of this study was to investigate the factors associated with increased operation mortality and morbidity, and to identify the relationship between gender and surgical outcomes of CABG surgery among the patients. We analyzed a single center's CABG surgery outcomes in Iranian men and women until the patients were discharged from the hospital.

## 3. Patients and Methods

### 3.1. Patient Population

We prospectively studied 690 consecutive adult patients (495 men and 195 women) who underwent isolated CABG with cardiopulmonary bypass (CPB) in the Rajaie Cardiovascular Center, Tehran, IR Iran over a period of five months. Patients having concomitant valve repair or replacement were excluded. Population breakdown by gender was performed to obtain two groups of men and women. Preoperative, intraoperative, and postoperative variables, as well as major complications, including death, were gathered and compared between the men and women until their discharge from hospital. Preoperative and operative clinical variables were prospectively gathered and evaluated including; sex, age, body mass index (BMI), smoking history, opioid addiction, arterial partial pressure of oxygen (PaO_2_), clinical class by New York Heart Association (NYHA) functional class, ejection fraction (EF), diabetes mellitus, renal failure, timing of surgery, and reoperation. Diabetes was defined in terms of treatment with oral hypoglycemic agents or insulin. Timing of the operation was designated as elective or urgent (acutely unstable patients were those who came from the CCU or catheterization laboratory). Information included; notes obtained from the operating room, operation time, pump time, cross-clamp time, number of grafts, and internal mammary use. Anesthetic induction was performed using; benzodiazepine, thiopental, fentanyl or sufentanil, and non-depolarizing muscle relaxants. Maintenance of anesthesia was continued with isoflurane, midazolam or Propofol, sufentanil and non-depolarizing muscle relaxants. The internal mammary artery was used preferentially for the left anterior descending coronary artery. Saphenous vein grafts were used in all patients. The number of saphenous vein and arterial bypass grafts used in the course of the operation were also determined.

In the postoperative period, exudation criteria and ICU discharge were the same for all of the study patients. During the ICU stay period, we collected the frequency of major complications such as; postoperative myocardial infarction (MI), inotropic requirements, bleeding, respiratory complications, renal failure, neurological complications (transient ischemic attack, stroke), infections, and in-hospital deaths. MI was defined as a new Q wave in at least two leads of an ECG or creatine kinase levels (CK)-MB at more than twice normal levels or 10% of total CK. Postoperative inotropic requirement was defined as any inotropic drug requirement to maintain hemodynamically stability for more than one hour. Respiratory complications were defined as; plural effusion requiring thoracocentes is or atelectasis. Postoperative renal failure was defined as an increase in creatine levels of more than 1 mg/dL above the preoperative value. Transient ischemic attack was defined as focal neurological deficit. Stroke was defined as a new and permanent global or focal neurological deficit. Infections, including sternal wound infections, were diagnosed if antibiotics therapy was required. In-hospital mortality was defined as a death which occurred during or after cardiac surgery in the hospital stay period.

### 3.2. Statistical Analysis

Initially, statistical analysis was performed with SPSS 15.0 for Windows (SPSS Inc. Chicago, IL, USA) and this analysis was used to identify and compare the risk factors between men and women. In the second stage, to minimize confounding between the genders, we adjusted for age and diabetes, which appeared to be the main risk factors among the female patients, and the effect of sex, was examined again. Women were compared to men using independent sample t-tests for the continuous variables or a chi-square test (or Fisher's exact test) for the category variables. Continuous variables were presented as mean ± standard deviation and category variables as a number (percentage). Results were considered statistically significant at P value ≤ 0.05 levels.

## 4. Results

### 4.1. Pre-Operative and Operative Variables

Of the 690 patients included in this study, 28.3% were women and 71.7% were men. The preoperative and operative characteristics of the women and men are listed in Table 1 and 2. Compared with the men, the women were older and had a higher incidence of diabetes, higher body mass index (BMI), lower preoperative PaO_2_ and lower functional capacity by the NYHA functional classification. Men were more likely to have poorer ventricular function and a significant history of tobacco use. The incidence of previous chronic obstructive pulmonary disease COPD and renal dysfunction were the same. There were no differences between; emergency or reoperation cases, operation time, CPB time, and aortic cross clamp time. During the surgery, women received the same number of total grafts, but fewer arterial grafts.

**Table 1.** Analysis of Preoperative Patient Characteristics by Sex

Variables	Women, N = 195	Men, N = 495	*P* value
Age, year	59 ± 8^a^	57 ± 10	0.020
BMI^b^	28 ± 4.5	27 ± 4	0.011
NYHA^b^ functional class			0.0001
Ι, ΙΙ	140 (71.5 %)	412 (83.3 %)	
ΙΙΙ, ΙV	55 (28.5 %)	83 (16.7 %)	
Ejection fraction			0.030
< 35	17 (9 %)	64 (13 %)	
35-49	70 (36 %)	210 (42 %)	
≥ 50	108 (55 %)	22 (45 %)	
Diabetes mellitus	89 (45.6 %)	98 (19.8 %)	0.0001
COPD^b^	6 (3 %)	13 (2.6 %)	0.750
Renal failure	3 (1.5 %)	19 (3.8 %)	0.120
Smoking	7 (3.6 %)	191 (38.6 %)	0.0001
PaO_2_^b^,mmHg	71.2 ± 11.6	75.7 ± 11	0.003

^a^ Data are presented as mean ± SD

^b^ Abbreviations: BMI, body mass index; COPD, chronic obstructive pulmonary disease; PaO_2_, arterial partial pressure of oxygen; NYHA, New York Heart Association

**Table 2.** Analysis of Intraoperative Patient Characteristics by Sex

Variables	Women, N = 195	Men, N = 495	*P* value
Emergency case	7 (3.5 %)	14 (2.8 %)	0.602
Reoperation	2 (1 %)	11 (2.2 %)	0.310
Operation time , min	227 ± 52^a^	231 ± 53	0.299
CPB^b^ time , min	87.1 ± 29	86.8 ± 29	0.906
Aortic clamp time , min	48.1 ± 20	47.8 ± 19	0.838
Number of coronary grafts			0.240
One	6 (3.1 %)	13 (2.6 %)	
Two	36 (18.7 %)	85 (17.2 %)	
Three	113 (57.9 %)	253 (51.1 %)	
Four	40 (20.3 %)	100 (29.1 %)	
IMA^b^ use	183 (93.8 %)	483 (97.5 %)	0.016

^a^ Data are presented as mean ± SD

^b^ Abbreviations: CPB, cardiopulmonary bypass; IMA, internal mammary artery

### 4.2. Post-Operative Variables

Table 3 shows the postoperative variables and complications between men and women. Women had a higher incidence of pulmonary complications, and were more likely to require postoperative inotropic support. They had a higher incidence of decreased EF compared to their preoperative values. The incidence of postoperative arrhythmias and bleeding were similar in both groups. There were no differences between the two groups for; cerebrovascular accidents, transient ischemic attacks, renal failure, infections, or length of ICU stay. After discharge from the ICU, women had a greater incidence of CCU admission and the women also stayed in the hospital longer than the men. However, the incidences of return to ICU, and in-hospital mortality rates were similar for men and women.

**Table 3.** Analysis of Postoperative Patient Characteristics and Complications by Sex

Variables	Women, N = 195	Men, N = 495	*P* value
Ventilation time , hour	12.5 ± 20.7^a^	11.3 ± 20	0.483
Pulmonary complications	34 (17.4 %)	43 (8.6 %)	0.001
Arrhythmia	18 (9.2 %)	33 (6.6 %)	0.250
Inotropic use	29 (14.8 %)	47 (9.4 %)	0.031
Postoperative MI^b^	21 (10.7 %)	10 (2 %)	0.0001
Postoperative EF^c^			0.018
≥ preoperative EF	175 (89.7 %)	469 (94.7%)	
< preoperative EF	20 (10.3 %)	26 (5.3 %)	
Bleeding	25 (12.8 %)	74 (14.9 %)	0.470
Renal failure	4 (2 %)	5 (1 %)	0.280
Neurologic event	8 (4.1 %)	11 (2.2 %)	0.170
Infection	5 (2.5 %)	4 (0.8 %)	0.067
ICU stays, day	3.8 ± 4	3.4 ± 3	0.260
CCU admission	31 (15.9 %)	51 (10.3 %)	0.038
Hospital stays , day	12.2 ± 5	10.7 ± 3.6	0.006
In-hospital death	5 (2.5 %)	11 (2.2 %)	0.832

^a^ Data are presented as mean ± SD

^b^ Abbreviations: MI, myocardial infarction; EF, ejection fraction

^c^ Postoperative EF ≥ preoperative EF: Postop. EF equal or more than preop. EF, postoperative EF < preoperative EF: postop. EF less than preop. EF

Table 4 and 5 compares male and female subgroups selected by age and diabetes. In the adjusted groups, non-diabetic women > 50 years of age had more pulmonary complications and higher levels of post-operative MI (Table 4). The perioperative variables in the non-diabetic patients did not differ significantly between the women and men with ≤ 50 years of age (Table 5). In the diabetic patients (both > 50 and ≤ 50 years old subgroups) there were no statistically significant differences between the male and female patients.

**Table 4.** Comparison of Clinical Variables between Non-diabetic Male and Female Patients Aged > 50

Variables	Women, N = 87	Men, N =292	*P* value
BMI^b^	27.5 ± 4.5^a^	26.9 ± 4.2	0.300
NYHA^b^ functional class (/)			0.001
Ι, ΙΙ	60 (67.8 %)	250 (85.6 %)	
ΙΙΙ, ΙV	27 (32.2 %)	42 (14.4 %)	
EF^b^			0.160
< 35	7 (8 %)	33 (11 %)	
35 - 49	30 (35 %)	125 (43 %)	
≥ 50	50 (57 %)	134 (46 %)	
PaO_2_^b^ (mmHg)	71 ± 12	75 ± 9.6	0.048
IMA^b^ use	81 (93.1%)	286 (98.0 %)	0.024
Pulmonary complications	12 (13.8%)	18 (6.1 %)	0.020
Inotropic use	10 (11.5 %)	24 (8.2 %)	0.350
Postoperative EF			0.440
≥ preoperative EF	81 (93.1 %)	278 (95.2 %)	
< preoperative EF	6 (6.9 %)	14 (4.8 %)	
Postoperative MI^b^	6 (6.9 %)	6 (2 %)	0.040
CCU admission	11 (12.6 %)	30 (10.3 %)	0.502
Hospital stays , day	12.2 ± 5.8	10.7 ± 3.3	0.020

^a^ Data presented as mean ± SD

^b^ Abbreviations: BMI, body mass index; NYHA, New York Heart Association; EF, ejection fraction; PaO_2_, arterial partial pressure of oxygen; IMA, internal mammary artery; MI, myocardial infarction

**Table 5.** Comparison of Clinical Variables between Non-diabetic Male and Female Patients Aged ≤ 50.

Variables	Women, N = 19	Men, N = 105	*P* value
BMI^b^	28 ± ^a^ 4.3	26.9 ± 4.6	0 090
NYHA^b^ functional class			0.350
Ι, ΙΙ	13 (68.4 %)	86 (81.9 %)	
ΙΙΙ, ΙV	6 (31.6 %)	19 (18.1 %)	
EF^b^			0.270
< 35	0 (0 %)	11 (11 %)	
35-49	9 (47 %)	45 (45 %)	
≥ 50	10 (53 %)	49 (49 %)	
PaO_2_^b^(mmHg)	75 ± 8	79 ± 12	0.470
IMA^b^ use	16 (84.2 %)	102 (97.1 %)	0.016
Pulmonary complications	2 (10.5 %)	7 (6.6 %)	0.060
Inotropic use	2 (10.5 %)	8 (7.6 %)	0.650
Postoperative EF			1.000
≥ preoperative EF	18 (94.7 %)	100 (95.2 %)	
< preoperative EF	1 (5.3 %)	5 (4.8 %)	
Postoperative MI^b^	0	2 (1.9 %)	0.330
CCU admission	4 (21 %)	12 (11.4 %)	0.270
Hospital stays, day	11.4 ± 3.6	10.2 ± 2.4	0.220

^a^ Data are presented as mean ± SD

^b^ Abbreviations: BMI: body mass index; NYHA, New York Heart Association; EF, ejection fraction; PaO_2_, arterial partial pressure of oxygen; IMA, internal mammary artery; MI, myocardial infarction

## 5. Discussion

Previous studies investigating the influence of sex on mortality and morbidity following cardiac surgery have produced different results. Therefore, it remains difficult to determine whether gender has an important influence on cardiac surgery results, as we could not conclude that female gender, per se, is an independent predictor for cardiac surgery results. Several studies have revealed that the risk profile of men and women is quite different (2, 6, 7, 10, 12), so it may be quite inappropriate to attempt a comparison between men and women as the common risk factor. Numerous studies have shown that perioperative complications are related to comorbid risk factors, but not to female gender itself (15, 16). Some reports found a greater number of comorbid conditions and risk factors including; stroke, heart failure, diabetes, and higher NYHA functional class, particularly in young women (15). The findings of the present study confirm the results of some previous studies (13, 15-19). Kim et al. (20) in a systematic review of randomized clinical trials from 1995 to 2005 found that early mortality differences (higher in women) were reduced, but not consistently eliminated, after adjustment for comorbidities.

In our study, women represented 28.3% of the patients. In our research, the women were older and had more comorbid conditions and risk factors at surgical presentation, compared with the men. However, if the number of coronary artery grafts were identified as all diseased arteries, then the extent of multivessel disease were similar in both groups of patients. Women had better preoperative ventricular function, although their NYHA functional class was higher. Women also had more postoperative complications and longer hospital stays. Despite these differences, there was no statistical difference in the incidence of postoperative in-hospital deaths. Even after adjustment for these two risk factors, female sex remained independently associated with more complications among patients > 50 years of age, and sex differences in morbidity remained markedly different among female patients > 50 years of age.

The analysis of data from a single academic institution like our institution is associated with inherent bias. The main disadvantage of such studies is an inadequate sample size to distinguish differences in rare clinical variables between the study groups. In this study the lack of statistical significant differences between diabetic men and women could be due to the relatively small sample size of these groups of patients. On the other hand, a single institutional study has some advantages such as; uniform clinical practices, and it utilized the same members of the medical treatment team, who met the same criteria for patient care.

In summary, the present study showed that there were increased postoperative complications in women, in part, this was related to a higher incidence of diabetes and advanced age in this group of patients, and female gender may be an independent predictor of postoperative complications, as has been shown in multiple previous studies. Despite these differences, in-hospital mortality was not influenced by sex. Better preoperative left ventricular EF in the women and patient selection could probably explain this result. However, further studies are encouraged with higher numbers of patients to determine the level of support for these results.
